# Self-reported oral health outcomes after switching to a novel nicotine pouch technology: a pilot study

**DOI:** 10.2340/aos.v84.43805

**Published:** 2025-05-27

**Authors:** Giusy Rita Maria La Rosa, Karl Fagerström, Sebastiano Antonio Pacino, Jan Kowalski, Renata Górska, Stefan Gospodaru, Gheorghe Bordeniuc, Valeriu Fala, Amaliya Amaliya, Iain Chapple, Riccardo Polosa

**Affiliations:** a Department of Clinical and Experimental Medicine, University of Catania, Catania, Italy; b Fagerström Consulting, Stockholm, Sweden; c Addendo srl, Dental Clinic, Catania, Italy; d Department of Periodontology, Medical University of Warsaw, Warsaw, Poland; e Faladental, Chișinău, Republic of Moldova; f ’Nicolae Testemiţanu’ State University of Medicine and Pharmacy, Chişinău, Republic of Moldova; g Department of Periodontology, Faculty of Dentistry, Universitas Padjadjaran, West Java, Indonesia; h Periodontal Research Group, Institute of Clinical Sciences, College of Medical & Dental Sciences, The University of Birmingham, Birmingham, UK; i Birmingham Community Healthcare NHS Foundation Trust, Birmingham, UK; j Faculty of Medicine and Surgery, ‘Kore’ University of Enna, Kore, Italy; k Center of Excellence for the Acceleration of Harm Reduction (CoEHAR), University of Catania, Catania, Italy

**Keywords:** Nicotine pouch, oral health, smoking cessation, snus, tobacco harm reduction

## Abstract

**Objective:**

Nicotine pouch use has been linked to oral health concerns, including oral lesions and gingival irritation. This pilot study examines self-reported oral health outcomes following the use of a novel nicotine pouch (Stingfree Strong Blue Mint), with an impermeable barrier on the interior side designed to reduce mucosal irritation.

**Materials and methods:**

A total of 23 Swedish dentists who were current snus or nicotine pouch users participated in a 5-week observational study. Baseline and follow-up assessments included self-reported oral health status and photographic documentation of mucosal conditions, reviewed by an independent blinded dental expert. Primary outcomes included changes in self-reported snus lesions, gingival recession, gingival irritation, and gingivitis.

**Results:**

The prevalence of self-reported snus lesions decreased from 95.7% (*n* = 22) to 69.6% (*n* = 16). Median Axell-scale lesion severity declined from 2 (interquartile range [IQR]: 1–3) to 1 (IQR: 0–2) (*z* = 3.756, *p* = 0.0002). Moderate-to-severe lesions (Axell score ≥ 3) dropped from 39.1% (*n* = 9) to 0% (*n* = 0). Self-reported gingivitis cases (*n* = 3) were eliminated, and gingival irritation decreased by 90.0%.

**Conclusions:**

Preliminary findings suggest that the use of the Stingfree Strong Blue Mint nicotine pouch may reduce mucosal irritation. While promising, these findings warrant validation through large randomised controlled trials to establish long-term effectiveness and safety.

## Introduction

The health risks of cigarette smoking are well documented, with serious diseases such as lung cancer, cardiovascular disease, and chronic obstructive pulmonary disease linked to exposure to the various toxic substances produced during tobacco combustion. Cigarette smoke contains more than 7,000 chemicals, many of which are classified as harmful and potentially harmful constituents (HPHCs) [1]. However, these risks stem primarily from combustion by-products rather than nicotine itself, which mainly accounts for the addictive component of smoking [2, 3].

Oral nicotine pouches (ONPs) represent alternative nicotine delivery methods that are smokeless in nature and are placed under the lip for nicotine absorption. By eliminating combustion, they significantly reduce or eliminate exposure to HPHCs found in cigarette smoke [4–7]. A similar product, snus, has been available in Sweden for decades as a popular alternative to cigarette smoking [8–10]. However, unlike ONPs, snus contains tobacco. ONPs consist of a nicotine-infused cellulose matrix inside a small fibre pouch. The nicotine can be either naturally or synthetically derived. Although absorption varies based upon individual usage, the nicotine release from ONPs is expected to be similar to that of snus [11, 12]. While both product categories play a role in tobacco harm reduction (THR), nicotine pouches offer a tobacco-free alternative, appealing to those seeking a nicotine experience without tobacco-derived compounds.

The growing use of traditional snus and ONPs has raised concerns about their potential effects on oral health. Despite being marketed as less harmful alternatives to combustible tobacco, research suggests that these products may still contribute to adverse oral health outcomes, including mucosal lesions, gingival recession, and gingival irritation [13–15]. The severity of these effects varies based on factors such as pH levels, nicotine concentration, and frequency of use [16, 17]. Both traditional snus and ONPs have been linked to oral mucosal irritation and inflammation, particularly at the placement site [17, 18].

To address these concerns, a novel nicotine pouch technology, the Stingfree technology pouches have been developed with an impermeable barrier for protecting the gum to minimise direct gingival irritation [19]. Designed in a slim format for discreet and easy placement under the lip, these pouches replace tobacco with a blend of tobacco derived nicotine, salt, flavourings, thickeners, fillers, sweeteners, stabilisers, acidity regulators, water, and cellulose fibres. The impermeable barrier, composed of partly (50%) plant-based, renewable materials, is intended to reduce mucosal irritation while preserving sensory experience and nicotine delivery.

Given the increasing adoption of nicotine pouches and the limited research on harm reduction innovations in this category, it is important to examine potential oral health implications arising following their use. This pilot study provides preliminary insights into the self-reported oral health outcomes of Stingfree Strong Blue Mint. Over a 5-week period, the study assessed changes in snus lesions, gingival recession, gingival irritation, and gingivitis. Findings from this study will provide pilot data to help generate hypotheses to guide future larger, more rigorous randomised controlled trials (RCTs) on the potential benefits of this novel nicotine pouch technology.

## Materials and methods

This pilot study used a pre-post observational design to assess self-reported oral health outcomes among Swedish dentists after switching to Stingfree Strong Blue Mint, a novel nicotine pouch product. Individuals eligible for the study were required to be at least 25 years old, be regular users of snus or nicotine pouches, and interested to switch to a reduced-irritation nicotine pouch (Stingfree Strong Blue Mint) for 5 weeks. Participants received only the necessary quantity of Stingfree Strong Blue Mint products for the duration of the trial, with no financial compensation provided. Given the study’s focus on consumer product evaluation with self-reported outcomes, it did not require formal ethical approval. However, all participants provided informed consent, and measures were taken to ensure data protection and confidentiality.

To ensure maximum transparency and reliability, the specifications of the Stingfree Strong Blue Mint product had been verified through independent laboratory testing. The nicotine content is 12 mg/g, with a pouch weight of 0.55 g and a nicotine content per pouch of 6.6 mg. The water content, determined using the Car Fisher method, is 30 g/100 g, and the pH of 8.8 according to Coresta method No. 69, 2021. These measurements were confirmed by the independent Eurofins Laboratory in Sweden, which collaborates with most Swedish snus producers to measure and certify these values.

The study lasted 5 weeks and aimed to evaluate changes in snus lesions, gingival recession, gingival irritation, and gingivitis prior to and following exclusive use of the new product. Volunteers were recruited through two closed Facebook groups for Swedish dentists, where an initial call for participation was posted. Eligible participants were required to be at least 25 years-of-age, be current users of snus or nicotine pouches, and commit to using only Stingfree Strong Blue Mint for the study duration. A total of 44 dentists initially expressed interest, with 26 commencing the study and 23 completing it. Participants who withdrew cited reasons such as concerns about high nicotine strength or non-compliance with the exclusive use requirement.

Baseline data collection involved a self-administered questionnaire assessing participants’ oral health status, snus or nicotine pouch usage, and experiences of oral irritation.

Each participant received a supply of Stingfree Strong Blue Mint boxes (cans), each containing 20 nicotine pouches, corresponding to their self-reported average weekly usage, multiplied by 5 to cover the 5-week duration of the test, plus an additional 5 boxes. If a participating dentist declared using 5 boxes per week on average, he/she has then received 5 boxes × 5 weeks = 25 boxes, and an additional 5 boxes to ensure the products last during the full 5 weeks test period.

Although photographic documentation relied on participant initiative, detailed instructions were provided to standardise image acquisition. Participants were encouraged to use a clinic system camera or a high-resolution smartphone camera, and to photograph their usual snus placement location, ensuring that the lesion was fully visible and captured as perpendicularly as possible. They were also advised to use a lip retractor, chin retractor, or two dental mirrors when feasible, and to include the entire lesion – even the inner lip or side of the cheek, if applicable. Participants were instructed to maintain their usual oral hygiene practices throughout the study to minimise potential confounding factors. At the end of the 5-week period, participants completed a follow-up questionnaire replicating the baseline assessment and submitted new photographic documentation under identical conditions. The primary outcomes of interest included changes in the presence and severity of snus lesions, gingival recession, gingival irritation, and gingivitis, with lesion severity assessed using the five-degree Axell-scale [20–22]:

- Degree 0 indicates the absence of any oral lesions.
- Degree 1 corresponds to a superficial lesion with a colour similar to the surrounding mucosa, exhibiting slight wrinkling but no obvious thickening.
- Degree 2 is characterised by a superficial, whitish or yellowish lesion with wrinkling but no visible thickening.
- Degree 3 presents as a whitish-yellowish to brown wrinkled lesion with intervening furrows of normal mucosal colour and noticeable thickening.
- Degree 4 represents the most severe form, with a pronounced yellowish to brown heavily wrinkled lesion, deep reddened furrows, and/or significant mucosal thickening.

To ensure independent evaluation, all submitted photographs were randomised and reviewed by a blinded external dentist with no affiliation to the product manufacturer. The assessor evaluated lesion severity using the Axell scale and, when necessary, requested additional images to clarify unclear documentation. In cases where his evaluation differed from participants’ self-reported assessments, discussions were held, and adjustments were made where appropriate. No financial compensation for participants was provided.

### Statistical analysis

Descriptive statistics were used to summarise participant characteristics and oral health outcomes at baseline and follow-up. Median and interquartile ranges (IQRs) were reported for lesion severity due to the ordinal nature of the Axell-scale. A *per protocol* analysis was undertaken, rather than *intention-to-treat.* Changes in expert-reviewed lesion severity scores between baseline and follow-up were analysed using the Wilcoxon signed-rank test, a non-parametric method for comparing paired ordinal data. To assess whether baseline lesion severity was predictive of follow-up severity, an ordered logistic regression model was employed, treating both variables as ordinal. The proportional odds assumption was tested using the Brant test, confirming the validity of the model. Data analysis was performed using STATA/BE v.17 statistical package (StataCorp LT, College Station, TX, USA). Statistical significance was set at *p* < 0.05.

## Results

Of the 26 participants who initially enrolled, 23 successfully completed the 5-week study. Three volunteers withdrew due to concerns over nicotine strength or because of non-compliance with the exclusive product use requirement. The final sample comprised of 20 males and three females, with a broad age range of 25 to 65 years (mean age = 42.8 years). The duration of snus and/or nicotine pouch use among participants varied. The majority (43.5%, *n* = 10) reported using these products for more than 20 years, 17.4% (*n* = 4) reported a usage period of 10 to 20 years, and 30.5% (*n* = 7) had been using them for 5–10 years. In addition, a smaller proportion of participants (8.7%, *n* = 2) had used snus and/or nicotine pouches for 5 years or less.

The majority of participants (82.6%, *n* = 19) reported exclusive use of snus and/or nicotine pouches at baseline. A smaller number of participants also reported using other nicotine products. Specifically, 8.7% (*n* = 2) reported cigarette smoking, 4.35% (*n* = 1) used e-cigarettes (vapes), and another 4.35% (*n* = 1) consumed other nicotine-containing products. The median consumption of oral pouches among participating dentists was four cans per week, aligning with typical usage patterns in Sweden, with some consuming up to nine cans per week.

Baseline assessments indicated that most participants reported oral health concerns related to snus or nicotine pouch use, including mucosal lesions, gingival irritation, and gingival recession. Among the 23 participants who completed the study, baseline lesion severity varied between the two groups. Exclusive snus users had a higher median lesion severity score (median = 2, IQR = 1) compared to exclusive nicotine pouch users (median = 1, IQR = 1). Similarly, the mean lesion severity score was higher among snus users (mean = 2.18, standard deviation [SD] = 0.75) than nicotine pouch users (mean = 1.58, SD = 1.08). However, these differences were not statistically significant (*t*(21) = 1.53, *p* = 0.142; Mann-Whitney U test: *z* = 1.71, *p* = 0.0868).

After 5 weeks of exclusive use of Stingfree Strong Blue Mint, notable reductions were observed in the prevalence and severity of snus-induced oral lesions and gingival irritation. Snus lesion prevalence declined from 95.7% (*n* = 22) to 69.6% (*n* = 16), with lesion severity significantly reduced over 5 weeks. The median Axell-scale lesion severity score decreased from 2 (IQR: 1–3) to 1 (IQR: 0–2), confirmed as statistically significant (Wilcoxon signed-rank test; *z* = 3.756, *p* = 0.0002). A total of 15 participants (65.2%) experienced lesion improvement, while eight (34.8%) remained unchanged, and none worsened. Ordered logistic regression analysis indicated that baseline lesion severity significantly predicted follow-up lesion severity (OR = 2.25, 95% CI: 1.05–4.82, *p* = 0.037).

Furthermore, the proportion of participants with moderate-to-severe lesions (Axell grades 3–4) decreased from 39.1% (*n* = 9) at baseline to 0% post-study, indicating a clinical improvement of the most severe cases. Self-reported gingivitis cases, present in 13.0% (*n* = 3) at baseline, were completely absent post-study, while cases of gingival irritation declined by 90%, from 43.5% (*n* = 10) to 4.3% (*n* = 1). Gingival recession, however, remained unchanged, with 39.1% (*n* = 9) of participants reporting this condition before and after the study (see Table 1).

**Table 1:** Changes in self-reported oral health outcomes before and after using Stingfree Strong Blue Mint.

Oral health condition	Baseline % (n)	Post-study % (n)	% Change
Snus lesions (any severity)	95.7% (22)	69.6% (16)	↓ 27.3%
Axell-scale lesion severity (mean score)	2 (IQR: 1–3)	1 (IQR: 0–2)	↓ Significant (*p* = 0.0002)
Gingival recession	39.1% (9)	39.1% (9)	No Change
Gingival irritation	43.5% (10)	4.3% (1)	↓ 90.0%
Gingivitis	13.0% (3)	0% (0)	↓ 100.0%

IQR: interquartile range.

Although no formal subgroup analysis was conducted, participants who used nicotine pouches only (*n* = 12) experienced a 46.2% reduction in snus lesion grade after switching to Stingfree Strong Blue Mint for 5 weeks. Similarly, those who used snus only (*n* = 4) demonstrated a 37.5% reduction. Despite the unequal group sizes, a comparable trend of lesion improvement was observed.

**Figure 1:**
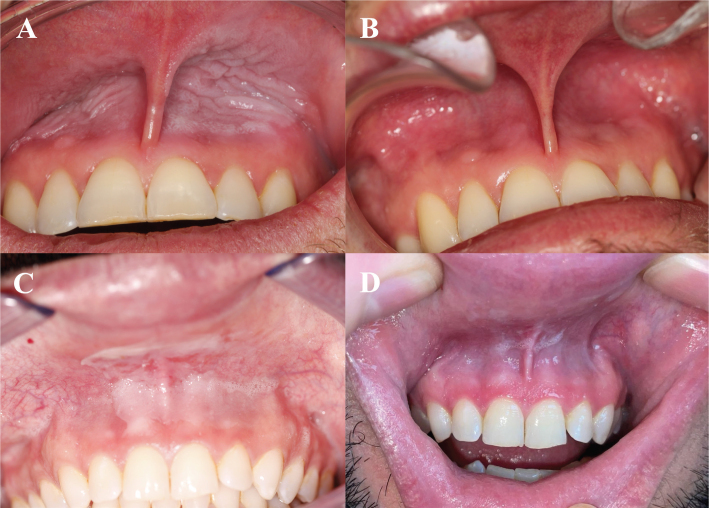
Snus lesion severity before and after a 5-week trial. (A) Severity 3/4 before trial in a 40–45-year-old male dentist using snus and nicotine pouches for 20+ years. (B) Severity reduced to 0/4 after 5 weeks, with a total of 45 cans consumed (9 per week). (C) Severity 4/4 before trial in a 35–40-year-old male dentist using nicotine pouches for 5–10 years. (D) Severity reduced to 1/4 after 5 weeks, with a total of 35 cans consumed (7 per week).

Visual analysis of participant-submitted photographs con-firmed a decrease in snus lesion severity, with no remaining cases of Axell-scale grade 3 or 4 lesions at 5 weeks (Figure 1). Amongst participants who initially presented with moderate-to-severe lesions (grades 3–4, *n* = 9), all experienced improvement, with none remaining in the highest severity categories after 5 weeks of product use. While most participants reported positive outcomes, one individual noted increased oral dryness compared to his regular nicotine pouch brand. No participants reported worsening mucosal irritation or the development of new lesions during the study.

## Discussion

This pilot study provides preliminary insights into the potential effects of Stingfree Strong Blue Mint on self-reported oral health outcomes amongst Swedish dentists who use nicotine pouches or snus. The findings suggest a reduction in the prevalence and severity of snus-induced mucosal lesions, with a 27.3% decrease in lesion occurrence and a significant reduction in lesion severity. In addition, cases of gingivitis were eliminated, and reports of gingival irritation declined by 90% over the 5-week study period. These trends suggest that the impermeable barrier within Stingfree nicotine pouches may reduce mucosal irritation compared to traditional nicotine pouches, aligning with the proposed mechanism of protection [19]. However, due to the lack of a control group, these findings should be interpreted with caution as external factors, such as changes in user behaviour, cannot be ruled out. Moreover, the available evidence on the regeneration of snus-related lesions in smokers and non-smokers is poor. Further studies are needed to better understand the factors influencing these processes.

A notable finding of our study is that no participant experienced an increase in lesion severity, and cases classified as Axell-scale grades 3–4 at baseline improved to lower scores (grades 0–2) after 5 weeks of using the Stingfree Strong Blue Mint pouches. None remained in grade 3–4 in the Stingfree study. In agreement, a recent study reported the prevalence of white mucosal lesions decreased to 70% among the participants that following the replacement of Swedish snus with NP [16]. In contrast, Miluna et al. [23] found that most participants using snus or nicotine pouches developed white oral mucosal lesions of varying textures and shapes. The study also linked these changes to the duration and frequency of tobacco use, with those consuming 5–10 units daily or using these products for 5–10 years more prone to such lesions. However, a major limitation of their study is that it is difficult to separate Swedish snus users from nicotine pouch users, making it challenging to directly compare their results with those of the present study. This finding underlines the need for further research to explore potential factors contributing to lesion regression and persistence among nicotine pouch users. In addition, while these results suggest that the Stingfree pouch may reduce mucosal irritation, a direct comparison with other nicotine pouches is necessary to determine whether its impermeable barrier provides superior protection against lesion development.

Further analysis using ordered logistic regression confirmed that baseline lesion severity was a significant predictor of follow-up lesion severity. This suggests that participants with higher baseline severity scores were more likely to have persistent lesions at follow-up, despite the overall reduction in lesion prevalence and severity. While this finding does not contradict the overall improvement seen in the study, it indicates that pre-existing severe lesions may require longer exposure to the product to fully resolve. This highlights the importance of longer follow-up periods in future research to determine whether extended use leads to further improvements.

One of the key observations in this study was that gingival recession remained unchanged, despite reductions in other indicators of oral irritation. This suggests that while the novel nicotine pouch design may alleviate short-term mucosal irritation, it may not directly influence periodontal effects such as gingival recession, at least in the short term. Gingival recession is a clinical condition that can be managed through various treatment approaches such as periodontal plastic surgery, however, it is not a condition that can naturally reverse on its own [24, 25]. In addition, while self-reported gingival irritation decreased substantially, one participant noted increased oral dryness, highlighting the need for further exploration of user experiences beyond lesional healing alone. A recent systematic review found no serious adverse events associated with nicotine pouches [17], with Alizadehgharib et al. [16] reporting only mild-intensity events, including dry mouth. However, the review included only three studies [16, 23, 26]. This aspect, alongside the general acceptability of the product, needs to be addressed in future studies to better assess potential side effects and user satisfaction.

Finally, recent studies have suggested that certain flavours and additives in ONPs may trigger toxicological responses with prolonged use [27, 28]. For this reason, the impact of flavourings in products like Stingfree warrants further investigation in future research.

A key strength of this study is its novel focus on a nicotine pouch product designed to minimise oral irritation, an area with a limited existing research base. The use of self-reported outcomes supplemented by photographic assessments provides a valuable combination of subjective and objective data. In terms of feasibility, the study had high adherence, with 23 out of 26 participants completing the study.

Some limitations remain to be addressed. Although this was an exploratory pilot study, the small sample size, limited observation period, and absence of a control group should be acknowledged as potential limitations. Future studies with larger sample sizes and longer follow-up periods, including appropriate control groups, are recommended to better assess the long-term effects and the stability of the observed improvements. The potential bias introduced by self-reported outcomes is mitigated, in part, by the use of photographic documentation reviewed by a blinded assessor and by the fact that, as oral health professionals, dentists routinely diagnose oral lesions in patients who use snus or nicotine pouches – products regularly consumed by approximately 20–25% of the adult population in Sweden. This professional familiarity may help reduce bias in their self-assessments. Nevertheless, self-reporting bias cannot be entirely excluded, and the findings should therefore be interpreted with appropriate caution. Unlike previous research [16], which found a statistically significant correlation between the extent of product use and lesion improvement, our study was unable to assess this relationship as all participants adhered fully to their exclusive use of Stingfree Strong Blue Mint. Future studies with larger participant numbers and with levels of adherence may help determine whether the intensity of product use influences the extent of mucosal healing. Finally, the sample consisted predominantly of male participants, which may affect the generalisability of the findings.

Research on the impact of ONPs on oral health is currently scarce [17, 29]. A recent RCT evaluated the impact of switching from smoking to nicotine pouches on oral health in adult smokers. The study reported a significant reduction in signs of gingival inflammation and bleeding, suggesting that nicotine pouches may help mitigate the oral health risks associated with traditional smoking [30]. Data specific to the impact of the Stingfree pouch on oral health are lacking. Future studies should incorporate larger, more diverse populations to validate these preliminary results and determine their generalisability. A RCT comparing Stingfree pouches to traditional nicotine pouches or snus is necessary to establish causality in reducing oral irritation. Long-term studies are also needed to evaluate the progression of gingival recession and whether prolonged use of the product offers sustained benefits for oral health. In addition, further research should explore other potential effects, such as changes in salivary pH, microbial composition, and user satisfaction, to provide a more comprehensive understanding of the role of Stingfree in harm reduction strategies.

Nicotine pouches may represent a viable alternative within THR strategies, particularly in dental settings, where prof-essionals should be equipped to understand both the risks and potential benefits of these products [31].

## Conclusion

This study provides preliminary evidence that the Stingfree Strong Blue Mint nicotine pouch may reduce snus-induced mucosal lesions and gingival irritation. While self-reported outcomes suggest improvements, the lack of a control group and small sample size limit definitive conclusions. The high adherence rate and combination of self-reported and photographic data confirm the feasibility of further research. Future studies should focus on RCT designs with a broader population to establish causality and assess long-term effects. These findings serve as a foundation for future research on nicotine pouch innovations and harm reduction strategies in oral health.

## Data Availability

The data that support the findings of this study are available from the corresponding author, upon reasonable request.
